# Common opossum (*Didelphis marsupialis* Linnaeus, 1758): food and medicine for people in the Amazon

**DOI:** 10.1186/1746-4269-10-65

**Published:** 2014-09-10

**Authors:** Flávio Bezerra Barros, Pierre de Aguiar Azevedo

**Affiliations:** Programas de Pós-Gradução em Agriculturas Amazônicas (PPGAA) e Antropologia (PPGA), Universidade Federal do Pará. Avenida Augusto Corrêa, N° 1, Cidade Universitária “José da Silveira Netto”, Guamá, CEP, 66075–110 Belém, Pará Brazil; Faculdade de Ciências Sociais. Universidade Federal do Pará, Instituto de Filosofia e Ciências Humanas, Avenida Augusto Corrêa, N° 1, Cidade Universitária “José da Silveira Netto”, Guamá, CEP, 66075–110 Belém, Pará Brazil

**Keywords:** Ethnozoology, *Didelphis marsupialis*, Traditional Medicine, Hunting, Amazon rainforest

## Abstract

**Background:**

In the Amazon rainforest, biodiversity is a significant resource for traditional communities, as it can be used as a relevant source of protein and it has a promising zootherapeutic potential. Studies on knowledge and ways how local peoples use the fauna are still incipient. This paper presents both the knowledge on and food and medicinal uses of common opossum (*Didelphis marsupialis*) by riverine communities in an Amazon floodplain region.

**Methods:**

The study was conducted with riverine communities in the municipality of Abaetetuba, Pará, Brazil. The main methods used were structured and semi-structured interviews, the “snowball” technique, and participant observation.

**Results:**

The study showed that *D. marsupialis* has an undeniable cultural significance for the local community, both in terms of food and medicine. Its meat is prized by inhabitants as it is classified as tasty, soft and, in some cases, it is designated as the best bushmeat in the region. The interviewees have demonstrated a thorough knowledge on various aspects of the animal’s biology, such as its diet, behavior, and places of occurrence. The hunting activity is practiced by men, but the preparation of meat and medicinal oil are tasks mainly performed by women. In medical terms, common opossum is used in the treatment of various diseases, such as rheumatism, asthma, sore throat, and inflammation. Given the importance of this species, its meat or live individuals are often sold in the city fair at prices that can reach R$ 40.00 (U$D 18,00) per individual.

**Conclusions:**

*D. marsupialis* is an important source of protein for riverine communities in the region studied. Its fat is used as a traditional medicine and it is indicated for many types of diseases. Although the species concerned is treated with hostility in various Brazilian regions, in the case of Abaetetuba this animal is strongly prized due to the good quality of its meat. However, despite the value assigned to the species, its consumption should be the subject of further studies, as this marsupial species has been described as a reservoir for parasites that cause severe diseases.

## Background

The Amazon rainforest, which is among the largest biodiversity reserves in the world, plays an important role in the life of traditional communities, providing food, housing, transportation, household items, furniture, income, remedies, as well as other environmental services. Accordingly, the hunted fauna is crucial both as a protein and remedy source. Several studies conducted around the world have documented, through Ethnozoology, knowledge and ways how human populations use animal species [1–7]. Despite the biological and cultural richness of the Brazilian Amazon rainforest, studies addressing this subject of the Ethnozoology [8–18] are scarce, considering the variety of uses of fauna adopted by human communities in the region. Indeed, in Brazil, studies on Ethnozoology are more advanced in the Northeast region [5, 19, 20].

This article presents the results of a survey carried out with riverine populations of the Amazon floodplain in the municipality of Abaetetuba, Pará, Brazil. Populations inhabiting islands in this town often use the marsupial species *Didelphis marsupialis* Linnaeus, 1758 (Mammalia: Didelphidae), a mammal with a wide geographical distribution in the Americas [21] that occurs from Mexico to Argentina; it is culturally significant in the region concerned. This marsupial species is among the most adapted and widespread Neotropical mammals, occurring both in forest areas and in urban environments, with great plasticity in its diet, consuming fruits, small animals, and, when occurring in urban environments, it can feed on garbage [21, 22].

Our interest in conducting studies on this species is mainly due to the fact that, in other Brazilian regions, including the Amazon rainforest, this animal is treated with hostility by people, often being killed. According to registered accounts, its strong odor (in Brazil, it is named “catinga” [nauseating odor] or “pitiú” [fish-like odor]) and the habit of preying upon eggs and poultry are the main factors that contribute to this kind of reaction. Some of the previous studies reported food and medicinal uses of this species [12, 13, 17, 23–28], and it is also present in myths and beliefs of some indigenous and traditional peoples [28–30]. Another important element of this species is that it is a reservoir for various parasites, which can cause health problems for human beings [31, 32]. This paper aims to present ethnoecological knowledge and the main uses of common opossum by riverine communities in Abaetetuba, Pará, Brazil, and think through the risks of eating this species posed to human health.

## Materials and methods

### Study area and local community

**Figure 1:**
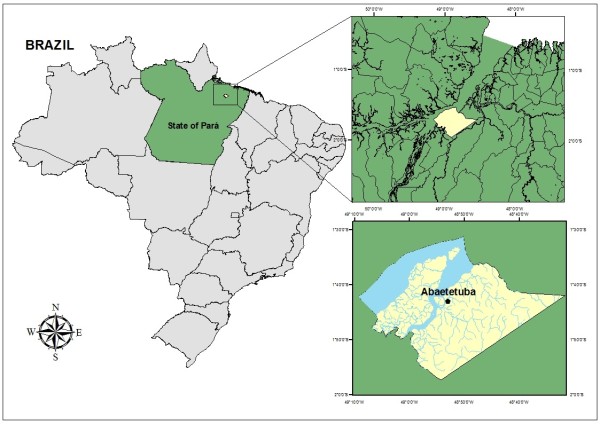
**Map of the study area.**

The study was conducted in the Community “Maracapucu Sagrado Coração de Jesus”, between November/2012 and March/2013, in Abaetetuba, Pará, Brazil (Figure 1). According to Monteiro [33], the word *abaetetuba* comes from the Indigenous language Tupi, and it means “gathering of strong and true men”. The town is about 60 km away from the capital city of Pará, Belém, it has an archipelago consisting of 72 islands and an area that comprises both rural and urban zones, with a population of 141,100 inhabitants, distributed over a total area of 1,610,606 km^2^ [34]. The community is spread across 3 islands along the Maracapucu River: Nazaré, Quianduba, and Guajará, about 30 minutes away from the town hall, by “rabeta”, an engine-powered type of boat. The prevailing environment in this area is the floodplain, an ecosystem typical of the Amazon characterized by a seasonally flooded lowland forest. Seasons are well defined, with summer usually occurring from June to November and winter from December to May. Average temperature in the region is around 25°C.

The region is also characterized by several myths, such as the “boto myth”, a cetacean species that inhabits the Amazon rivers. In this town, inhabitants tell that the aquatic animal usually charm girls and carry them to the bottom of rivers. Another version says that it comes in the form of a handsome young man dressed in white to charm girls during the night, usually at parties, and impregnates them, disappearing soon after. For details about stories involving the “boto”, see Slater [35]. Another important story in the region is the myth of “Great Snake”. According to the local population, this supernatural being inhabits the bottom of Maratauíra River and a part of the town. Residents say that its head is in the Church of Our Lady of Conception, located downtown, its body stretches by the river (Maratauíra River, which bathes the town), and the tail is underneath Pacoca island, one of less inhabited islands in the region; certainly, because of “Great Snake”, which sometimes moves under the river and, when angry, it even breaks sidewalks in the town pier.

**Figure 2:**
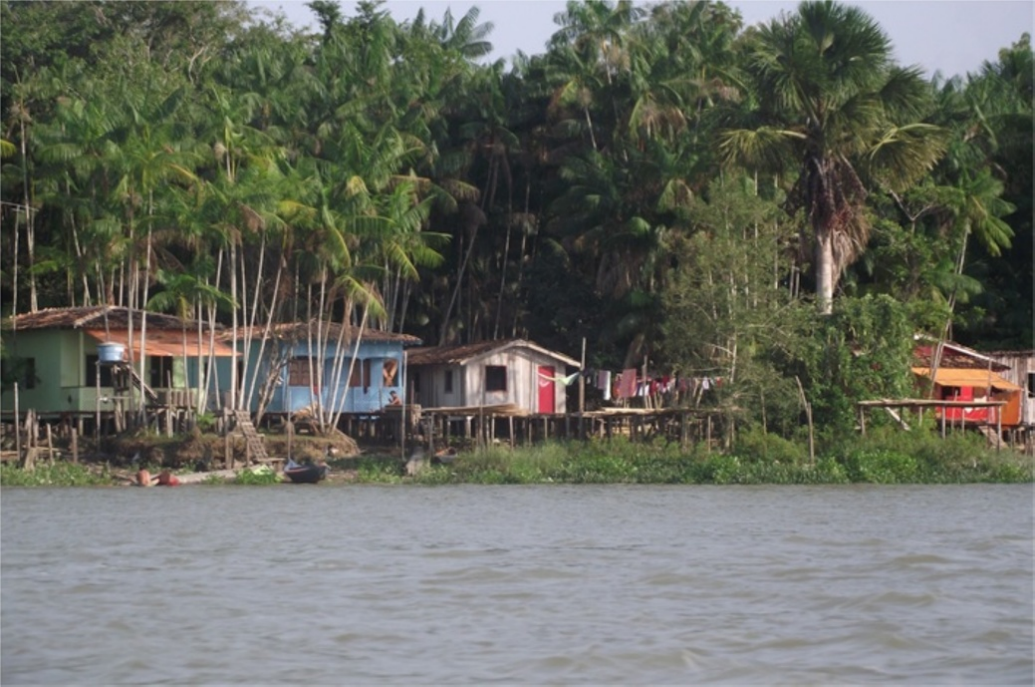
**Riverine house in Abaetetuba, Pará State, Brazil.** Photo: PAA.

Currently, about 130 families distributed over the 3 islands constitute the riverine community; people inhabit the river banks, where they build wooden houses, at a distance that water cannot reach during high tide (Figure 2). The main productive and economic activities for this population are: hunting animals, such as anteaters, agouti, opossum, racoon; fishing for several fish and shrimp species; and vegetal extraction, mainly from açaí palm (*Euterpe oleraceae* Mart.) and moriche palm “miriti” (*Mauritia flexuosa* Mart.). They practice subsistence agriculture, by growing vegetables, fruit trees, and manioc (*Manihot esculenta* Crantz), and also handicraft, such as “miriti toys”, “matapi”, basketry, etc.; everything is made of raw material from Amazon palm trees. In the community, there is only one public Elementary School, which is located in Guajará island, and a boat transports children. The predominant religions are the Catholic and Evangelical; however, there are records of “Pajelança” in this region, a shamanic healing practice whose origin dates back to the beliefs and customs of the ancient Tupinambá Indians, syncretized through contact with white and black people; it has a strong connection to nature and disembodied entities [36–38].

### Methods

We conducted expeditions to the town at different times, covering the winter and summer seasons. This dynamics allowed us to observe the way how riverine people interact with the environment at different seasons by using various natural resources found in the region. Relying on contact with a local leadership, who is also a member of the Association of Rural Workers in the town, we had access to the other respondents participating in the survey. This procedure led to greater commitment and trust between us and the community. In a meeting with the community, research objectives were duly introduced and all participants signed the free and informed consent term.

The survey was conducted by means of methods from Anthropology and Ethnoecology, such as participant observation [39, 40], which consists in interaction between the researcher and inhabitants, through participation in everyday life activities. This method allowed us to know, at a deeper level, key elements of lifestyles in the local community. All information obtained through observation was registered in a field diary.

Through the first interviewee, we had access to all other actors, using the so-called “snowball” method, which consists in the last interviewee indicating an expert on the subject within the community to participate in the study [41]. Altogether, we interviewed 18 people, 15 men and 3 women, aged from 26 to 74 years, distributed into the islands: Nazaré (n = 9); Guajará (n = 5); and Quianduba (n = 4) (see Table 1). Out of this total, in 4 interviews there was voluntary participation of a second family member. In order to address data as a whole, we considered all information on the subject under investigation, even when it was reported by only one interviewee [42], combining various competences. We conducted semi-structured and structured interviews, following the recommendations made by Huntington [43], and informal conversations also took place [44].

**Table 1:** **Information about respondents (age, gender and locality) and ethnoecological information about common opossum/respondent (weight, breeding season, number of young/pregnancy, food diet and habitat)**

Respondents	Age	Gender	Locality (Island)	Weight (Kg)	Breeding season (number of times/year)	Number of young/pregnancy	Food diet	Habitat
1	55	M	Nazaré	2 - 5	February to May (3)	4-8	Fruits	Canopy “cofó” of miriti palm
2	38	F	Guajará	2	- (-)	-	Fruits	Hollow tree
3	67	M	Nazaré	2	June (2-3)	8-9	Fruits	Canopy “cofó” of miriti palm
4	74 (47)	F (M)	Guajará	2-3	June to August (-)	5-6	Fruits	Miriti palm and hollow tree
5	46	M	Guajará	2-3	August (1)	2-7	Fruits	Floodplain
6	62	M	Nazaré	1,5-4	August (3)	4-8	Fruits	Miriti palm and river edge
7	27	M	Nazaré	1-2,5	- (-)	-	Fruits	-
8	52 (28)	M (M)	Nazaré	2-4	November to January (1)	3-6	Fruits	Canopy “cofó” of miriti palm
9	53 (54)	M (F)	Nazaré	1,5-4	February to May (1)	4-7	Fruits	Ground, canopy of miriti palm, river edge
10	58	M	Quianduba	1-4	- (-)	4-6	Fruits	Canopy of miriti palm and hollow tree
11	60	M	Quianduba	1,5-4	August to January (1)	7	Insects, hen, eggs,	Canopy of miriti palm and hollow tree
12	64	M	Quianduba	1-4	- (-)	4-6	Fruits and insects	Canopy of miriti palm and hollow tree
13	45	F	Nazaré	1-3	June to December (-)	5-6	Fruits	Canopy of miriti palm and hollow tree
14	26	M	Nazaré	1-3	August to September (2)	5-13	Fruits	Canopy of miriti palm and hollow tree
15	62	M	Guajará	2-5	All year (muny)	3-7	Fruits	Canopy of miriti palm and hollow tree
16	38	M	Guajará	2-5	January to March (3)	7-10	Fruits and crustaceans	Canopy of miriti palm and hollow tree
17	46 (36)	M (F)	Nazaré	2-3,5	December to March (1)	4-11	Fruits and hen	Canopy of miriti palm and hollow tree
18	40	M	Quianduba	1-4	All year (many)	3-12	Fruits	Canopy of miriti palm and hollow tree

Legend: F (Female); M (Male).

As the survey focused on analyzing ethnoecological knowledge and ways how riverine people use common opossum, there was a need to identify which species was regarded as the most important in the contexts concerned, since, through respondents’ reports and field observations, we registered various marsupial ethnospecies, which are popularly known in the region as “mucura” [opossum]. As we did not collect any zoological material, we used the literature currently available on the subject, such as Guide to Mammals from the Neotropical Forest [21], as well as the assistance provided by experts on the zoological group concerned, in order to make sure that the main species for the community was the common opossum (*D. marsupialis*). In the field, we used photos contained in Guide to Mammals…, so that riverine people could identify which ethnospecies occurred in the community and how they were named, because, locally, people usually add a second word to the term “mucura” opossum [black opossum], often an adjective that characterizes the animal. For each ethnospecies cited, we asked respondents to speak about morphological features, habits, and places of occurrence. For this procedure, we followed the recommendations made by Berlin [45]. Data were analyzed from an ethnographic perspective.

## Results and discussion

### Ethnotaxonomy and features of common opossum

Through the local ethnotaxonomy, respondents pointed out various types of opossum, which were distinguished considering their morphological features (body size, fur color, tail shape, eye shape, etc.), habits, and places of occurrence. We registered 5 ethnospecies of opossum, classified by respondents this way: “mucura preta” [common opossum], “mucura branca” [white opossum], “mucura morganha” [“morganha” opossum], “mucura xixica” [“xixica” opossum], and “mucura do fundo” [bottom opossum]. The occurrence of these ethnospecies in the region was supported by the taxonomy provided in the literature [21], geographical distribution data available on the website “Red List”, from the International Union for Conservation of Nature (IUCN) [46], and reports by specialized researchers. The species were: *D. marsupialis*, which corresponds to common opossum, *Caluromys philander* Linnaeus, 1758*, Chironectes minimus* (Zimmermann, 1780)*, Metachirus nudicaudatus* (E. Geoffroy, 1803), and *Philander opossum* (Linnaeus, 1758); however, we do not know if cited species correspond to ethnospecies informed by respondents, as we did not collect animals for identification. In fact, we do not mean that there are only such ethnospecies, but we do claim that they are those best known by riverine people, and common opossum (*D. marsupialis*) is the most important due to its food and medicinal value. Therefore, our study is exclusively devoted to this species.

Common opossum is a marsupial species from the Didelphidae family, known by different common names in the various Brazilian regions (“gambá” [opossum], “timbu” [white-eared opossum], “cassaco”, “sariguê”, “micurê”, “tacaca”, etc.). Female individuals from this group have a pouch named *marsupium*, used to carry offspring until they complete their development. Marsupials have nocturnal habits, they are usually solitary and provided with a defense mechanism that consists in expelling a fluid with a strong odor produced by a pair of perianal glands, which serves to ward off potential predators [21]. This feature ends up providing it with the bad reputation of a very dirty animal, besides being known as predator of poultry; thus, it is killed due to such behaviors. Even so, at certain locations in the Amazon rainforest, as in the case of Abaetetuba, Pará, the relationship between the animal and communities is relatively harmonious.

### Ethnoecological knowledge on common opossum

Common opossum, according to local actors, can be found with a weight variation between 1 and 5 kg (see Table 1), depending on the age group. This information is consistent with that present in the available literature [47, 48]. According to respondents, the species feeds primarily on fruits that occur in the region, such as “miriti” (*Mauritia flexuosa*), mango (*Mangifera indica* L.), Malay rose apple (*Syzygium malaccense* L.), açaí palm (*Euterpe oleracea*), genip (*Genipa americana* L.), besides insects, eggs of wild and domestic birds, including chicken. This omnivorous habit was observed in other studies, such as those carried out by Charles-Dominique [49] and Julien-Laferriere and Atramentowicz [50]. Waste-eating is a habit also documented in this species [21].

Regarding reproduction, the vast majority of respondents commented that common opossum is reproduced at different times of the year, but it is from June to August that the activity occurs with greater intensity. According to inhabitants, these periods are known as “vagrancy times - *vadiação*”. This information is consistent with results of studies on population density and reproductive activity of this species conducted in Venezuela [28] and Colombia [47], where authors indicated that *D. marsupialis* reproduces throughout the year.

However, some residents said that reproduction occurs every 6 months. Due to the high reproductive capacity and large number of young, riverine people often say that common opossum is a “mineral animal”, a local term that means good restoration capacity of the hunting resource in nature. Indeed, actors informed us that the animal reproduces from 1 to 3 times a year, usually within full moon periods, when residents observe individuals more frequently in “vagrancy – vadiação”. An interesting factor that was present in more than one report was that in August the opossums become ill, they face “liver problems”, and there are constant fights at this time of the year between individuals, as they are seeking for partners to reproduce. According to respondents, the amount of young can range from 2 to 13 individuals, which remain under mother’s care for a time that can vary from 2 to 4 months after birth. In a study on this species in southern Brazil, authors reported females ranging from 4 to 9, with an average of 6.5 young [51]. In surveys carried out in other countries in South America, the number of young ranged from 3 to 9 in Venezuela [28] and in Colombia the average number of young per female/year was 13.5 [47].

A key feature perceived by those who have a closer contact with common opossum is the fact that this species has a defense mechanism characterized by the production of a fluid with a strong odor, which is expelled against its predators. According to information provided by riverines, such fluid is produced by glands in armpits, locally named as “travancas”. The characteristic odor, known as “catinga” [nauseating odor], is frequently reported during “vagrancy-vadiação”, because females fight with other females when seeking for males to mate. Information about the location of these glands is different both from that available in the literature and that provided by experts, i.e. odoriferous glands are located in the perianal region instead of the underarms [21, 31].

### Strategies and times to hunt common opossum

Hunting is usually practiced by men and, on certain occasions, women accompany hunters. Knowledge of hunting strategies is orally transmitted from generation to generation, by fathers, uncles, or grandfathers. Many riverine people, still young, are taken to hunts by their fathers, who teach them how to become good hunters. The importance of teaching is primarily based on the idea that children do not go through hard times when food is scarce, being able to hunt for resources available in the region. Hunters’ training takes place gradually, as children grow, because the woods, according to respondents, pose many dangers, they are not safe for unaccompanied children. When the decision to hunt is taken, usually hunters prefer to take along a relative (brother, cousin, son, nephew, etc.) who is able to assist them in the undertaking.

**Figure 3:**
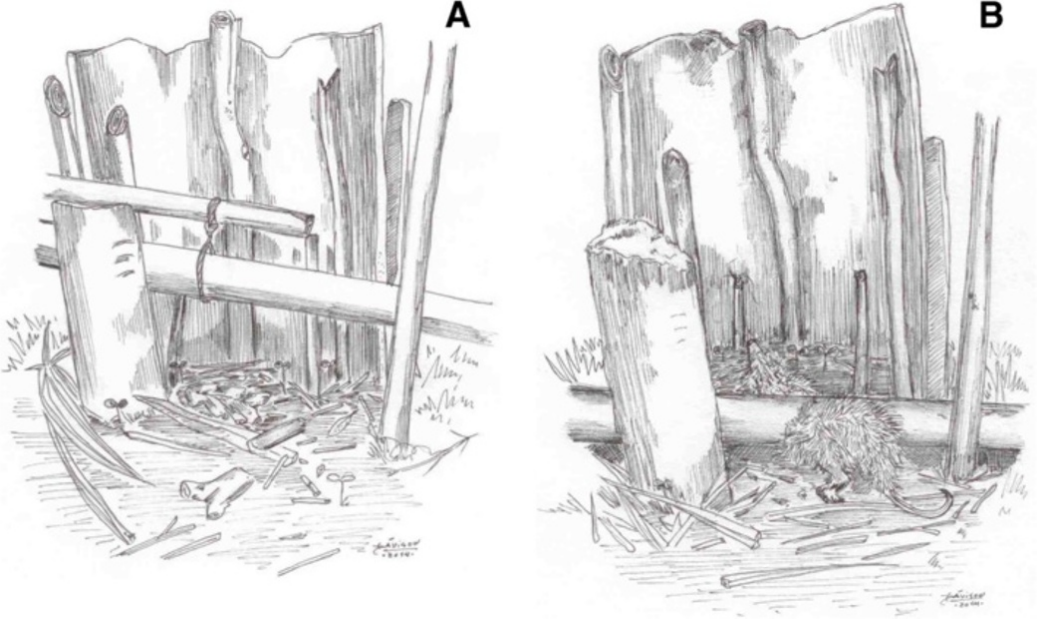
**Steps of the common opossum hunting with "Mundé" trap. A)** “Mundé”; **B)** Mundé with common opossum killed.

Riverines usually hunt common opossum by the morning or at night, using various techniques, among them traps, such as “mundé” (Figure 3A and B), which is built by hand, with raw materials found in the forest itself, such as pieces of wood and vines. The bait, usually prepared with a fruit having an attractive smell, is placed on the trap to lure the animal, which by eating activates the drop of a stick that kills it. However, this strategy is currently the least frequently used, because it does not allow the hunter to select by sex and age, nor identify pregnant females or those with offspring. Decreased use of this technique is associated with the idea of sustainability, because riverine people themselves said that “mundé” does not select animals and it is, therefore, harmful. As observed by a interviewee with regard to the use of this trickery: “*‘Mundé’ kills them all. What goes there, lies there*”.

**Figure 4:**
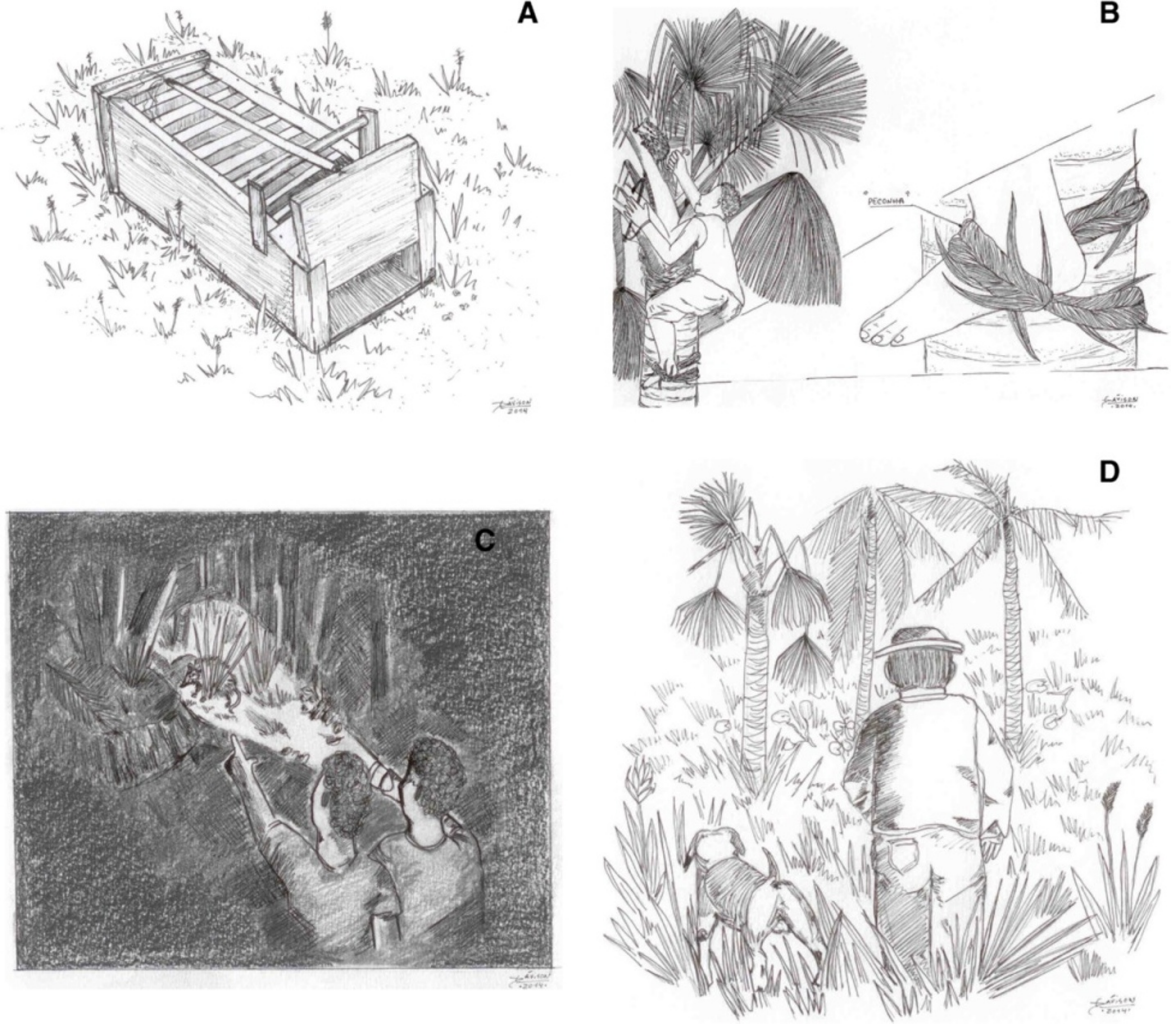
**Examples of techniques used to hunt common opossum. A)** Mousetrap to capture commom opossum; **B)** "Peconha" - is used to climb miriti palm; **C)** Flashlight - is used to illuminate the prey; **D)** Hunting with dog.

An alternative to using “mundé” is the mousetrap (Figure 4A), also produced by hand, but this kind of trap does not kill the animal, just catches it, giving hunters the chance to release the animal in case it does not meet the standard considered adequate for consumption. According to some actors, this notion of sustainability has been discussed at meetings of the Association of Rural Workers and other spaces that provide this kind of debate. In addition to these handicraft techniques, inhabitants use firearms, such as rifles, besides some cold weapons, such as the machete.“Peconha” (Figure 4B), a device that works as a belt holding feet, is made of plant fibers and intended to facilitate climbing trees up to the canopy where opossums are at rest. In this case, the catch is made with hunter’s hands or with the help of a hook that removes the animal from its burrow. And when the hunt takes place at night, a flashlight (Figure 4C) is used to illuminate the sites and enable viewing of the opossum.

Dogs are used in hunting excursions (Figure 4D), as they help sniffing out places where opossums hide and even capturing them, but certain residents noticed that the use of dogs must be done with caution, to prevent unnecessary killing of animals. The variation of techniques, used according to location, time, and hunter’s experience, demonstrates that traditional knowledge is not static, changes are based on people’s involvement in the environment to which they belong [52].

When asked about places where they are usually found, respondents informed us that common opossum inhabit different places according to the season. In summer, they are usually seen in the “cofó” (canopy) of trees, and during winter they are observed in hollow woods and in bushes. All these sites are occupied by the morning, when animals are resting, as they have nocturnal habits, when they leave to forage or, as locally said, “mariscar” [peck the ground]. During the hunting excursions, knowledge on the preferred environments of this animal greatly facilitates finding and capturing individuals when hunters rely on the use of dogs, which quickly identify a common opossum by sniffing.

Indeed, some hunters reported that common opossum, when faced with a hunter, stands watching him, motionless, trying to recognize its predator. When excursions are nocturnal, they are dazzled by flashlights and start fighting with the hunter, trying to scare and expel him. According to reports by riverine people, the best times to hunt a common opossum are by the morning, when they are resting in burrows and there is better light; or at night, when individuals are in “vagrancy” or “mariscando” [pecking the ground]. Actors also said that between 8:00 p.m. and 02:00 a.m. is an excellent time to hunt.

When the hunters have difficulties to obtain the game, a situation locally known as “panema”, they use various ways to reverse this failure state, such as, for instance, “bathing” the bandolier with chili pepper and then put it in a smokehouse. Many riverines reported us that pregnant women can cause “panema” if they eat bushmeat; in this case, there are a variety of bath recipes to drive misfortune away. Another practice taught was squeezing a lemon into the barrel and across the rifle, leaving it hanging down for two days at the pathway through which everyone passes, then the rifle must be washed in hot water. “Panema” is a state widely known among Amazon hunters, it is described in various anthropological and ethnoecological studies [8, 53–55].

When asked about the number of times per week they hunt, the answers were varied; there were respondents who hunt from 1 or 2 times/week to once a month. In the past, the hunting activity in the town was practiced more frequently, but nowadays, due to ease of access to processed foods or those derived from plant extraction and fishing, combined to better income and welfare policies adopted by the Brazilian government, we notice lower dependence on hunting as a primary source of protein. Studies conducted with traditional communities in other Brazilian regions have shown greater independence from forest resources for survival due to better income [56, 57]. However, it is worth highlighting that hunting remains as an activity practiced in this Amazon town, as *D. marsupialis* is among the few current alternatives [21].

Another situation observed was a hunting that happens when a relative, who does not live in the community, visits the family and takes the opportunity to hunt. According to some residents, on holy days and Sundays people cannot hunt, because these days must be reserved. Studying the hunting activity at the “Riozinho do Anfrísio” Extractive Reserve, in Pará, Brazil, Barros [55] has observed that riverine people usually reserve holy days and holidays to rest, they avoid hunting and any other kinds of work, because, according to residents, in these specific dates, the woods are inhabited by enchanted (or supernatural) beings, who protect animals from danger [58]. These supernatural beings are called “Curupira” (see figure in the cover) or they are generally named “Mãe-da-Mata” [Mother of the Woods].

**Figure 5:**
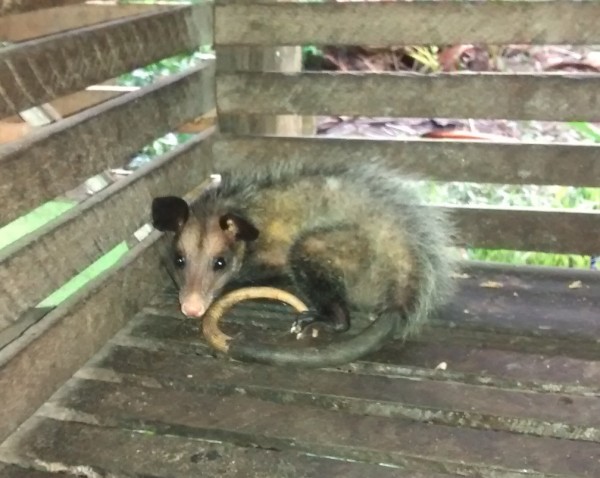
**Common opossum bred in captivity.** Photo: Roberta P. Fortes.

Regarding the best time to hunt, respondents answered that it depends on the season, taking into account the time of fruits and weather. During winter, from December to May, opossums are fatter, while in summer, from June to November, when there is less availability of food, animal population decreases. On the other hand, riverine people said that during summer capture is easier, as the ground is drier. It is worth taking this element into account, since the floodplain area, which undergoes seasonal flooding, when the soil is moist, hinders mobility both for residents and animals living in the area. They also reported that the hunter faces difficulties during full moon days, as he can be easily noticed by the animal, thus facilitating its escape. We can see that, regardless of the time of year when the activity is practiced, there is always a need to observe the various details in the environment. As we noticed during interviews and observations, respondents’ preference lies on male opossums, because they are fatter, they have more meat for consumption. In some cases, once captured, they are bred for a time to gain weight (Figure 5). Currently, hunting is practiced with a concern to avoid killing females, because, this way, they could contribute to the development of this animal, by not restricting reproduction.

## Uses of common opossum

### Food use: preparation and consumption

Regarding meat preparation, first, the fur is removed, something which may be done in two ways: by putting the animal in direct contact to fire or by leaving it in boiling water so that the fur is soften and it becomes easily removed. When it is a male, before slaughter, it must undergo castration to avoid “pitiú”, local term that means “stink”. After removing the fur, the animal must be cleaned, extracting parts that will not be used, for instance, “miudagem” (viscera). In locality all can to eat the meat no restriction. Fat is used to produce medicinal oil, something which is discussed below.

Meat was generally classified as having a very good flavor, when compared to other species, such as cattle, pig, and chicken. It was also characterized as very soft, it is similar to other bushmeats, such as lowland paca [*Cuniculus paca (*Linnaeus, 1766)], deer [*Mazama americana* (Erxleben, 1777*)*], and common agouti (*Dasyprocta aguti* Linnaeus, 1758). Respondent reports illustrate the importance of hunting for the local gastronomy:

*This meat is tenderer than beef. I think it is like the meat of common agouti, lowland paca, or deer.* (Antonio Dias, interviewed on 10 Dec. 2012).

*I like it a lot. It has a different flavor, it is very soft.* (Osvaldo Rodrigues, interviewed on 11 Dec. 2012).

*The meat is very tasty. When restful, it becomes better. I mean by restful meat that the animal did not undergo stress.* (Manoel Pereira, interviewed on 10 Dec. 2012)

According to respondents, glands producing the odoriferous fluid, 4 in total, are located close to the 4 animal’s limbs involved in locomotion; this information differs from that provided by Roque et al. [31], who indicate that these glands are located in the animal’s perianal region. According to respondents, removing these glands is a challenge during meat preparation, because not everyone knows the location and the way how collecting them. Thus, it is worth highlighting an issue. If our interviews showed that information provided by residents on the location of these glands are controversial, what do riverine people remove from the animal? Unfortunately, we could not observe how bushmeat was handled before cooking.

When cleaning, we can use lemon or garlic and vinegar, in order to eliminate “pitiú”. Meat may be roasted over a wood fire, baked, or fried in oil. Another detail highlighted during interviews was the fact that the animal must be prepared immediately after slaughter, at the risk of unpleasant flavor changes if it takes too long to be cooked. Indeed, when the common opossum is hunted at night, it is preferable to keep it alive until the next day. Certainly, change in meat flavor may be associated with odoriferous glands, as they are triggered as a result of stress to which the animal is subjected.

In fact, the stress of the chase was reported as a factor that influences on meat flavor, i.e. an animal that is slaughtered without stress will taste better than that which has been slaughtered under stressful conditions. Thus, “restful” meat, as riverine people say, is tastier. Comparing meat flavor to that of other species, we were informed that common opossum has a specific, unique, and incomparable taste. Some respondents prefer eating animals that live in the forest instead of those found in urban areas, as the latter usually feed on garbage [21, 28].

An important aspect to be mentioned with regard to the consumption of *D. marsupialis* is related to the fact that this wild mammal is reservoir for *Tripanosoma cruzi* Chagas 1909, the parasite causing Chagas disease [31, 59–61], which triggers heart failure and digestive problems and it can lead to death, if not treated. According to studies conducted by Roque et al. [31], also in Abaetetuba, several *D. marsupialis* individuals captured were contaminated with the parasite, thus constituting a public health problem. A study pointed out that, when the animal is handled before cooking, there is risk of infection with parasites, especially if its blood is contaminated. Other studies, carried out both in Brazil and in Colombia [32, 61], pointed out that *D. marsipialis* is also a reservoir for the parasite *Leishmania braziliensis guyanensis* Viannia 1911, which causes leishmaniasis, a disease that affects dogs and human beings and it triggers problems both in epithelial tissue and in viscera; the flagellate parasite *Tetratrichomonas didelphidis* (Hegner and Ratcliffe 1927) was observed, too [62]. Because of this evidence, it is worth discussing the use of *D. marsupialis* from a public health perspective, in face of the risk that local population is contaminated by eating opossum meat.

In the community under study, overall, respondents said that consumption takes place from 1 to 3 times per week; if on the one hand men are in charge of hunting, on the other hand, when it comes to preparing the delicacy, women take on the task, thus demonstrating a clear work division, as the process involved in bushmeat consumption has to be analyzed according to its different steps. Just as fathers teach their son hunting strategies, mothers, aunts, and grandmothers train their daughters to become good cooks of opossum meat; however, some men venture to prepare it, as reported by some respondents. Indeed, participant observation, by means of interaction with families, allowed us to understand how many daily tasks are accomplished. These observations showed us that work division is flexible in certain situations, i.e. both men and women may perform tasks that traditionally are not assigned to their gender, with the exception of hunting, an exclusive task of men.

### Medicinal use: medicine made of common opossum in the Amazon

When preparing the opossum meat, fat, locally known as “banha” [fat], is separated to produce the handmade medicinal oil. This medicine is produced by melting fat over a fire until it becomes oil. After this, a scent freshener is added to oil, to attenuate the strong smell, such as camphor, a substance extracted from the camphor tree (*Cinnamomum camphora* L.), a species from the Lauraceae family. The medicinal oil is used as an anti-inflammatory for muscle pains, rheumatism, bruises, asthma, but mainly for sore throat. It is also indicated for pregnant women, because, according to respondents, this oil eases childbirth pain. This indication is associated with the fact that female opossums, as pointed out by riverine people, do not feel pain while giving birth to their young, which complete their development in a pouch, the marsupium. According to local belief, opossums have received the blessing of Our Lady, the Mother of Jesus Christ, who, one day, when asking milk from a lactating woman, had her request denied; the opossum, in turn, by hearing such a denial, gave her milk to Our Lady and then received the gift of never feeling pain during birth. This is the cosmological explanation for the marsupium and the absence of pain among female opossums.

Studies conducted in other parts of the Amazon rainforest [12, 13, 17] have registered the use of opossum for food and medicinal purposes. Terra and Rebêlo [13], in the state of Amazonas, registered the use of *D. marsupialis* bile and tongue to alleviate pain and asthma in pregnant women, respectively. In the first case, the use is similar to that identified in this study, although the substance used is different. Other studies show that the species concerned is widely used in the Brazilian traditional medicine [24–26, 63, 64]. The use of animal fat for medicinal purposes has been registered in many countries, such as Nigeria [65], India [66], Mexico [67], Nepal [68], Argentina [69], among others.

The most appropriate time for applying the oil is at dusk or late at night, before going to bed, and it may be used 2 or 3 times a day, depending on the case. When asked about the effectiveness of this oil, people informed us that, besides being assured of its efficacy, they also think that faith is crucial, because believing in a higher power helps in achieving a successful outcome of the medicine. Another interesting account was the idea that “*every animal has a cure*”, i.e. every animal species has some curative property.

This tradition, even being practiced to a lesser extent today, is widespread among residents, and we realized that older individuals intend to keep using it, conveying knowledge to younger people, also when modern medicine is present. The relative ease of access to modern medicine and the better income enabled by policies adopted by the Brazilian government may explain the decrease in its use. Above all, women, recognize that such knowledge was acquired from elderly people, this is something learned “*watching while mom did that*”, as a female respondent said. The influence of modern medicine on health problems faced by residents has made the practice of traditional medicine to become less frequent in the region. This, mainly among older residents, has raised doubts concerning the use of synthetic drugs and the loss of traditional knowledge and practices.

### To obtain some income

Hunting opossum in the town is primarily motivated by meat consumption within the household and also to share it among neighbors and relatives, in order to strengthen ties of friendship and reciprocity [70]. However, some inhabitants hunt the animal for commercial purposes, as the meat of this marsupial species is prized by people living in the urban area [23].

In fact, people sell this meat in their own community or in nearby areas, on demand or not. In the town fair, which is located on the left bank of Maratauíra River, a respondent told us that, whenever he hunts, even without any order, it is relatively easy to sell the product in the town fair, because this meat is highly demanded by residents due to its flavor, considered very good. A respondent explained us that when açaí palm extraction (*E. oleraceae*) is low, hunting becomes an important income source. The price of a common opossum, which may be sold alive or already slaughtered, varies according to animal size and season of the year. It ranges from R$ 10.00 (U$D 4,50) to R$ 40.00 (U$D 18,00), when the animal weighs about 5 kg; it is worth highlighting that live individuals are more expensive. On average, a common opossum weighs 2 kg and it costs R$ 30.00 (U$D 13,50). As wild animal trade is illegal in Brazil, as provided by Law 9,605/1989 [71], usually hunters sell animals late at night, in the only town fair. In the rural zone, trade takes place in an overt way, because there is no inspection.

### Is opossum hunting sustainable?

Common opossum hunting, according to reports from some respondents, decreased when compared to the past, but it still plays an important cultural role in the lives of local actors. We did not conduct population studies with the species concerned nor measured the percentage of meat consumed daily by riverine people, but, taking into account the attitudes of some actors with regard to the hunting practice and changes in eating habits, we may claim that, seemingly, this activity has not caused a negative impact on the local populations of *D. marsupialis*. However, as for the size of animal population, we noticed controversial speeches among hunters.

Some respondents stated that there is a decreased number of animals, as a consequence of the increased human population and greater pressure on this resource, also for trade; other respondents argued that there is an increased number of opossums in the region. The latter ones ground their claim in the increased number of jobs and better income levels observed in recent years, factors that reduce the need for hunting. A third group said that the common opossum is a species that has a good reproductive capacity (it is a “mineral animal”), therefore, they think the population remains stable. Given the diversity of discourses, we realize that all reports must be considered, since each member of the community has a particular way of addressing the environment. So, there is a need for conducting studies on population dynamics, in order to determine the demographic status of this species.

Respondents’ concern about sustainability became clear when they reported to observe the species reproductive and growth periods, they claim to avoid hunting females with young. Another interesting discourse consists in observing the increased human population in the floodplain region and its consequent deforestation, something which led animals to get away from households. Thus, two respondents reported that opossums once came to the river bank and, nowadays, their sounds are no longer heard. This information reinforces the consequences of the relationship between community and environment: when deprived of their habitat, animals gradually move away from the households, avoiding a contact that could be harmful to their survival.

Another account was very important for understanding the relationship between human beings and nature in the community under study. A respondent said: “*If you bring everything you see, soon nothing else will be seen*”. This comment was made by a community leader on predatory hunting, which has been the key factor to explain a possible decrease in the number of opossums, as he found out that many hunters do not observe any evidence of animal reproduction and hunt both males and females. The same respondent told us that some hunters are proud to hunt a large amount of animals, even over twenty opossums per hunting expedition. He has already found dead opossums in the woods, which perhaps were regarded as small and not relevant for trade. On this subject, another community member said that: “*I think a person may hunt to sell a large amount of animals when he needs to do that for a living, but I do not agree with wastage, leaving bushmeat behind*”. This reinforces the idea of community sustainable hunting: people should avoid hunting a large amount of animals and local species may not be endangered, regardless of their current conservation status [46].

According to an interviewee, in addition to the precautions taken, as already reported in this article, guidelines were acquired from governmental institutions about sustainable hunting practices, in order to safeguard opossum reproduction and keep its current population. This reflects what Coelho-de-Souza et al. [72] pointed out as the joint management process involving governmental actors and civil society in the pursuit of a dialogue between knowledge as a tool for addressing biodiversity and appreciation of local community wisdoms and practices [72]. Although we have not enough data to claim that the hunting activity is sustainable in the region under study, given the controversial information provided by respondents, we have noticed the expression of a particular environmental rationale by some community members.

### Final considerations

Studies on Ethnobiology and Ethnoecology are of great relevance because they register knowledge of local peoples on environment and natural resources. Such knowledge is key to promote dialogue and a closer contact between researchers and local actors [73], encouraging processes to gather Brazilian State agencies and the traditional peoples who fight for their territory and access to natural resources. These processes must strengthen the cultural identity of peoples, as well as improve the dynamics of biodiversity conservation and management. This study pointed out that common opossum (*D. marsupialis*) constitutes a significant hunting resource for the local population, both from the food and medicinal viewpoint. In sporadic cases, it provides families with an additional income. The meat of this species is highly prized by residents, something which shows that hunting resources play an important role as a source of animal protein for traditional Amazon populations. Certainly, the pressure on *D. marsupialis* is due to shortage of other kinds of bushmeat in the region, as in upland forest areas, where there is a richer diversity of hunting fauna, people hardly eat opossum. We stress the importance of further research in the public health field, because some studies have pointed out the risks posed to human health by the consumption of marsupials contaminated with parasites.
